# Distal Airway Inflammation Is Linked to Small Airway Dysfunction in Asthma

**DOI:** 10.3390/medsci14020292

**Published:** 2026-06-05

**Authors:** Hà Pham-Ngoc, Thông Hua-Huy, Nhât-Nam Lê-Dông, Frédérique Aubourg, Stéfanie Habib-Maillard, Clémence Martin, Isabelle Honoré, Nicolas Roche, Anh Tuan Dinh-Xuan

**Affiliations:** 1 Department of Respiratory and Sleep Medicine, INSERM UMR1343, Assistance Publique—Hôpitaux de Paris, Cochin Hospital, Université Paris Cité, 75006 Paris, France; ha.phamngoc.cochin@gmail.com (H.P.-N.); huy-thong.hua@aphp.fr (T.H.-H.); frederique.aubourg@aphp.fr (F.A.); 2 Sunrise, 5101 Namur, Belgium; bacsinam81@gmail.com; 3 Department of Respiratory Medicine, APHP Centre, Institut Cochin (UMR 1016), Assistance Publique—Hôpitaux de Paris, Cochin Hospital, Université Paris Cité, 75006 Paris, France; stephanie.habib@aphp.fr (S.H.-M.); clemence.martin@aphp.fr (C.M.); isabelle.honore2@aphp.fr (I.H.); nicolas.roche@aphp.fr (N.R.)

**Keywords:** asthma, small airway dysfunction, airway inflammation, exhaled nitric oxide, alveolar NO concentration, blood eosinophils

## Abstract

**Background/Objectives**: Airway inflammation and small airway dysfunction (SAD) are key features of asthma. Inflammation can be assessed by blood eosinophil count (Eos) and exhaled nitric oxide (NO) parameters, including fractional exhaled nitric oxide (FeNO), bronchial NO flux (J’awNO), and alveolar NO concentration (CANO), the latter reflecting distal airway inflammation. We aimed to evaluate the association between Eos and the degree of airway inflammation as specified by exhaled NO parameters and to assess the relationships between exhaled NO parameters and small airway dysfunction using spirometric and plethysmographic indices. **Methods:** We conducted an observational study of asthmatic outpatients who underwent spirometry, plethysmography, and exhaled NO measurements. Multivariable regression models were used to estimate associations between Eos and FeNO, J’awNO, CANO, and spirometric/plethysmographic indices, adjusting for age, sex, body mass index, and relevant covariates. **Results:** The analytic cohort included 121 patients (49 men; 72 women; median age 54.2 years). Small airway obstruction and a range of airway inflammation severity were observed. Mean (SD) or median [IQR] values, as appropriate, were: FEF_75_ z-score −0.62 (0.96); FEF_25–75_ z-score −1.27 (1.29); RV z-score 1.26 (0.94); RV/TLC z-score 1.60 (1.10); J’awNO 60.20 nL/min (102.80); FeNO 23.61 ppb (37.61); CANO 3.80 ppb (4.12); and Eos 260 cells/µL (430). Log-transformed Eos (log[Eos]) was associated with FeNO, J’awNO, and CANO (adjusted marginal slope [95% CI]: 12.11 [9.35–14.69], 33.56 [25.34–41.19], and 1.14 [0.84–1.43], respectively). Log(Eos) was also positively associated with RV and RV/TLC, but negatively associated with FEF_25–75_, FEF_75_, and FEV_1_. Similarly, CANO was positively associated with RV and RV/TLC and inversely associated with FEF_25–75_ and FEF_75_. No significant associations were observed for FeNO or J’awNO. **Conclusions:** Blood eosinophils were independently associated with all exhaled NO parameters. The association between CANO and small airway ventilatory function indices supports a link between distal airway inflammation and SAD in asthma.

## 1. Introduction

Asthma is a major global health problem affecting more than 300 million people and characterized by chronic airway inflammation and variable expiratory airflow limitation and symptoms [1,2]. It is now well-established that asthma involves the entire bronchial tree, extending from the central bronchi to the lung periphery [3,4]. The small airways (non-cartilaginous airways < 2 mm in internal diameter), historically termed the “quiet zone” [5] because they contribute little to total airway resistance in healthy lungs, are a major site of inflammation and obstruction [6,7]. Small airway dysfunction (SAD) is highly prevalent across all degrees of asthma severity and is increasingly recognized as a key determinant of poor disease control, frequent exacerbations, and severe refractory phenotypes [6,8].

Despite the clinical importance of the distal lung, assessing SAD remains challenging because there is no single gold-standard diagnostic test [4]. While impulse oscillometry (IOS) and nitrogen washout testing are sensitive for detecting peripheral dysfunction, spirometry remains the most widely available and standardized technique in clinical practice [9,10]. Although FEV_1_ is relatively insensitive to early peripheral changes, mid-to-late expiratory flows, such as forced expiratory flow between 25% and 75% of forced vital capacity (FEF_25–75_) and forced expiratory flow at 75% of forced vital capacity (FEF_75_), have been shown to be valuable indicators of small airway obstruction [9,11,12]. Mechanistically, FEF_75_ captures late-expiratory flow at low lung volumes, when flow limitation is dominated by peripheral non-cartilaginous small airways that are prone to dynamic compression and premature closure, making it more sensitive to distal airway dysfunction than FEV_1_ or volume-averaged indices [13,14,15,16]. In addition, this premature airway closure and consequent air trapping, characteristic features of SAD, are reflected by residual volume (RV) and the residual volume to total lung capacity ratio (RV/TLC), making these parameters valuable indices in its clinical assessment [9,17,18].

Accurate assessment of airway inflammation is crucial for asthma management [19]. Blood eosinophil count (Eos) serves as a systemic marker of type 2 (T2) inflammation, and fractional exhaled nitric oxide measured at a standard flow of 50 mL/s (FeNO) is an established noninvasive biomarker of eosinophilic airway inflammation [1,20,21]. However, FeNO primarily reflects nitric oxide (NO) generated within the proximal conducting airways and may fail to detect inflammation in the peripheral lung [22]. To address this limitation, mathematical models of pulmonary NO exchange, most commonly the two-compartment model, partition exhaled NO into two flow-independent parameters: bronchial NO flux (J’awNO) and alveolar NO concentration (CANO) [22,23,24]. Validated in multiple studies, CANO is a model-derived parameter that has been investigated as a potential marker of distal lung inflammation, whereas J’awNO reflects proximal airway NO production [19,25,26].

The relationship between the distal inflammatory milieu and small airway dysfunction remains incompletely characterized in asthma. This study therefore aimed to investigate the associations between inflammatory biomarkers and small airway obstruction to improve understanding of the pathophysiology of the so-called silent zone in patients with asthma.

## 2. Materials and Methods

### 2.1. Study Design and Population

**Figure 1:**
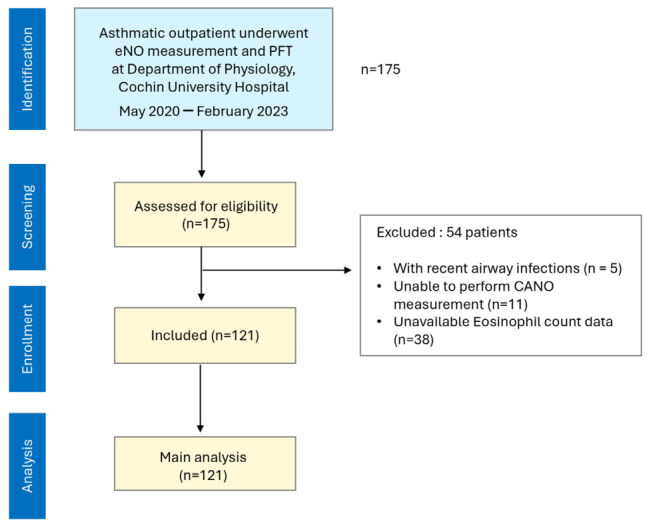
**Study flow chart.** *eNO: exhaled nitric oxide; PFT: pulmonary function testing; CANO: alveolar nitric oxide concentration (as measured by multi-sampling flow maneuver).*

We conducted an observational analysis of asthmatic outpatients who attended the Unit of Lung Function Testing, Department of Respiratory Physiology, Cochin University Hospital, Paris, France, between May 2020 and February 2023. A flow chart of participant recruitment is shown in the STROBE diagram (Figure 1). Inclusion criteria were age 18–85 years, physician-diagnosed asthma, and availability of spirometry, plethysmography, and exhaled NO measurements. Exclusion criteria were recent upper airway infection or pneumonia (<1 month), inability to perform CANO measurement (failure to obtain acceptable and reproducible multi-flow exhaled NO maneuvers due to technical failure, poor cooperation, air leak, cough, premature termination, or inability to maintain target expiratory flows), or unavailable Eos. All pulmonary function tests (PFTs), including exhaled NO measurements, were performed routinely after obtaining informed consent. The study complied with institutional regulations, and the need for additional written consent was waived by the local ethics committee.

### 2.2. Pulmonary Function Test

PFTs were performed using a MasterScreen Body system with SentrySuite software, version 3.20 (CareFusion, Höchberg, Germany) according to American Thoracic Society/European Respiratory Society (ATS/ERS) guidelines [27]. Global Lung Function Initiative (GLI) reference equations were used to calculate predictive values and z-scores [28,29]. Z-scores express the standardized deviations of measured PFT parameters from the corresponding GLI reference distributions, with negative values indicating lower-than-predicted measurements and positive values indicating higher-than-predicted measurements relative to the reference population.

### 2.3. Exhaled Nitric Oxide Measurement

Exhaled NO was measured before spirometry and before any bronchodilator administration during the pulmonary function visit using an electrochemical analyzer (FeNO^+^ Hyp’air, MGC Diagnostics Corporation, B-5503 Sorinnes, Belgium) in accordance with the ATS/ERS recommendations [29,30]. Standard pre-test instructions regarding food intake, caffeine, smoking, and strenuous exercise were applied. After a full inspiration to total lung capacity with NO-free air, subjects exhaled against the device while maintaining a positive mouth pressure between 5 and 20 cmH_2_O to ensure stable expiratory flows of 50, 100, and 150 mL/s. At each flow rate, at least two reproducible measurements (coefficient of variation < 10%) were obtained; a third measurement was performed when necessary. Mean NO output values at each flow rate were used to derive flow-independent parameters. CANO and J’awNO were calculated using the two-compartment model previously described by Tsoukias and George [24]. For each expiratory flow, NO output was estimated as the product of exhaled NO concentration and expiratory flow. CANO was estimated as the slope, and J’awNO as the intercept, of the linear relationship between NO output and expiratory flow (V’NO = J’awNO + CANO × V’E), using measurements obtained at 50, 100, and 150 mL/s. No additional axial diffusion correction was applied. FeNO and CANO are reported in parts per billion (ppb); J’awNO is reported in nanoliters per minute (nL/min).

### 2.4. Blood Eosinophil Count

Peripheral Eos were obtained from routine blood samples collected within 3 months of the pulmonary function and exhaled NO assessments. Eos were measured by standard automated differential cell counters and reported as cells per microliter (cells/µL). Eos values were log-transformed for statistical modelling when required because of skewed distributions.

### 2.5. Statistical Analyses

Analyses were performed with R software (version 4.3.1, R Foundation for Statistical Computing, 2023). Data are presented as mean (SD) or median [interquartile range, IQR] as appropriate, according to variable distribution. Associations between log-transformed eosinophil counts [log(Eos)], exhaled NO parameters (FeNO, J’awNO, CANO), and ventilatory function indices were assessed using multivariable regression models with appropriate distribution (Gamma for exhaled NO outcomes and normal for z-scores) and adjustments for sex, age, and body mass index (BMI). Results are reported as adjusted average marginal slopes, representing adjusted expected mean differences in the outcome per unit increase in the predictor of interest. Statistical inference was based on 95% confidence intervals (CIs) and two-sided null-hypothesis testing with a significance threshold of *p* < 0.05.

## 3. Results

### 3.1. Studied Population

Between May 2020 and February 2023, 121 consecutive adult outpatients with asthma and available exhaled NO, PFT, and Eos measurements were included (Figure 1). The cohort was middle-aged overall, with a median BMI in the overweight range; 57/121 patients (47.1%) were overweight, and 19/121 patients (15.7%) were obese. Most patients (92.6%) were not active smokers. All patients were receiving inhaled corticosteroid (ICS) therapy, 17/121 patients (14.0%) were receiving anti-leukotriene therapy, 34/121 patients (28.1%) were receiving antihistamines, and approximately one half (58/121, 48%) had an atopic background at the time of assessment. Relevant respiratory comorbidities included chronic obstructive pulmonary disease (8/121, 7%), rhinosinusitis (52/121, 43%), and bronchiectasis (14/121, 11.6%).

Nearly half of the patients (57/121, 47%) had Eos > 300 cells/µL. Exhaled NO measurements showed a gradient across flow-dependent and compartment-derived indices. FeNO at 50 mL/s was in the low-to-intermediate range (median value of 23.6 ppb) and decreased at higher expiratory flows (14.7 ppb at 100 mL/s, and 11.4 ppb at 150 mL/s). J’awNO remained high (median 60.2 nL/min; 5th–95th percentile range 16.3–266.5 nL/min). CANO was also elevated (median 3.8 ppb; 5th–95th percentile range 1.2–16.3 ppb). Mid- to late-expiratory flow indices were reduced on average, with negative mean z-scores for FEF_25–75_ and FEF_75_, whereas plethysmographic indices of air trapping were elevated, with positive mean z-scores for RV and RV/TLC (Table 1). To further characterize the burden of abnormal pulmonary function, categorized PFT outcomes are provided in Supplementary Table S1.

### 3.2. Relationships Among Systemic Eosinophilia, Exhaled NO Markers, and Small Airway Obstruction

**Figure 2:**
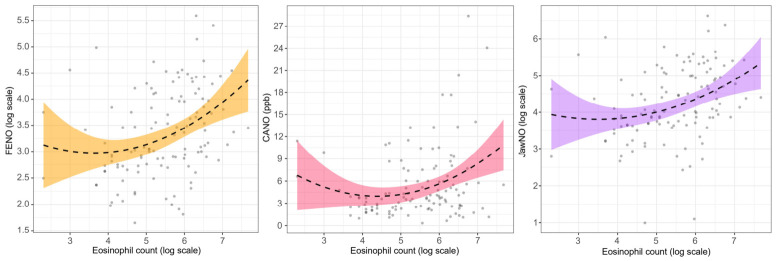
**Association between eosinophil count and exhaled nitric oxide markers.** *Regression plots illustrating the non-linear relationship between eosinophil count and the three main exhaled nitric oxide (eNO) markers: FeNO, CANO, and J’awNO. The x-axis shows log-transformed eosinophil count, and the y-axis shows the corresponding eNO outcome. FeNO and J’awNO were also log-transformed to stabilize variance. Dots indicate individual observations. The black curve represents the mean predicted value, and the shaded bands represent the 95% confidence interval, derived from a generalized linear model with a quadratic term for log-transformed eosinophil count. FeNO, fractional exhaled nitric oxide; J’awNO, maximal bronchial nitric oxide flux; CANO, alveolar nitric oxide concentration.*

After adjustment for covariates, higher log-transformed Eos [log(Eos)] was consistently associated with higher levels of all exhaled NO markers (Table 2, Figure 2). J′awNO showed the largest absolute marginal slope of 33.56 nL/min; (95% CI 25.34–41.19). Significant positive associations were also found for FeNO at 50 mL/s (adjusted marginal slope 12.11; 95% CI 9.35–14.69) and CANO (adjusted marginal slope 1.14 ppb; 95% CI 0.84–1.43]). In the complementary stratified marginal-slope analysis (Table S2), the associations between log-transformed eosinophil count and exhaled NO parameters were consistently stronger among patients with Eos > 300 cells/µL than among those with Eos ≤ 300 cells/µL.

**Figure 3:**
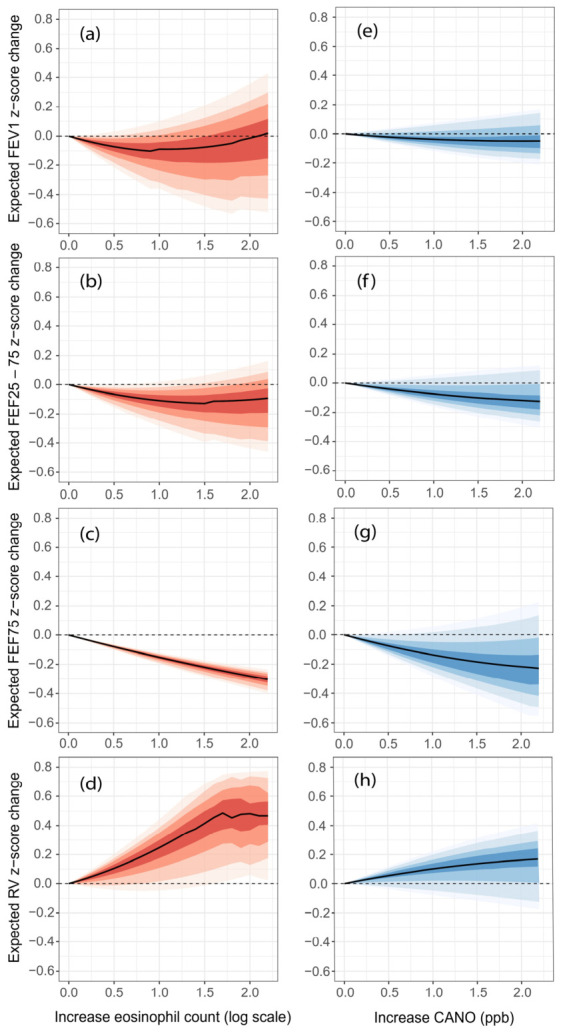
**Adjusted predicted changes in lung function z-scores according to eosinophil count and alveolar nitric oxide concentration.** *The graphs show the model-predicted change in pulmonary function z-scores associated with increasing eosinophil count ((**a**–**d**), logarithmic scale) and increasing CANO (**e**–**h**). The y-axis represents the adjusted predicted change in z-score relative to the reference value of the corresponding inflammatory marker. Negative predicted changes in FEV_1_, FEF_25–75_, or FEF_75_ indicate lower-than-expected airflow, whereas positive predicted changes in RV indicate greater air trapping. The solid line represents the average adjusted predicted change, and the shaded bands represent the uncertainty around the prediction. The horizontal dotted line denotes no predicted change. Models were adjusted for sex, age, and body mass index. FEV_1_, forced expiratory volume in 1 s; FEF_25–75_, forced expiratory flow between 25% and 75% of FVC; FEF_75_, forced expiratory flow at 75% of FVC; RV, residual volume; CANO, alveolar nitric oxide concentration.*

Regression analyses indicate that higher Eos were also associated with worse ventilatory function and greater gas trapping (Table 2, Figure 3). Specifically, increasing Eos were associated with lower FEF_25–75_, FEF_75_, and FEV_1_ z-scores, together with higher RV and RV/TLC z-scores; all associations were statistically significant. The stratified analysis (Table S2) suggested heterogeneity in the eosinophil–lung function relationship: associations with air-trapping indices, particularly RV and RV/TLC, were more pronounced at higher eosinophil levels, whereas associations with expiratory flow indices displayed a non-linear pattern across eosinophil strata. As shown in Table 3 and Figure 3, each 1 ppb increase in CANO was significantly associated with higher RV and RV/TLC z-scores and with lower FEF_25–75_ and FEF_75_ z-scores. In contrast, no significant associations were observed for FeNO or J’awNO. The association between CANO and FEV_1_ z-score was not statistically significant.

## 4. Discussion

We found that all exhaled NO parameters correlated positively with Eos, reinforcing their role as markers of T2 airway inflammation. The robustness of these associations is consistent with clinical guidance that identifies these measurements as validated noninvasive biomarkers of eosinophilic (T2) airway inflammation in asthma [20,31]. Elevated FeNO is characteristic of T2-high phenotypes and has been widely documented to correlate with eosinophilic airway infiltration [1,32,33]. Our data support the utility of combining Eos and FeNO to stratify T2 status, since these biomarkers reflect complementary aspects of the inflammatory cascade, including cytokine-driven eosinophil recruitment and inducible nitric oxide synthase (iNOS) expression [1,20,33,34,35].

J’awNO showed the largest marginal slope in relation to Eos (33.27), a finding consistent with prior work indicating that J’awNO primarily reflects NO production in the proximal conducting airways [25,36,37]. Importantly, we also observed a small but significant positive association between Eos and CANO (adjusted marginal slope = 1.14). Moreover, the Eos–CANO association was more pronounced among patients with Eos > 300 cells/µL and weaker at lower eosinophil levels. Whereas standard FeNO is dominated by NO produced in the proximal airway and may miss distal pathology, CANO is a distinct surrogate for inflammation in the peripheral airways and alveolar region [22,38,39]. This result reinforces earlier reports showing that CANO correlates with eosinophil counts in bronchoalveolar lavage (BAL) fluid but not necessarily with sputum or bronchial wash eosinophils [25]. Other studies have similarly reported elevated CANO in patients with eosinophilic asthma, particularly when small airway involvement may be missed by spirometry alone [39,40,41,42].

The correlation of Eos with both J’awNO and CANO suggests that systemic eosinophilia is associated with inflammatory activity throughout the bronchial tree, supporting the concept that partitioned exhaled NO parameters reflect the spatial extent of T2 inflammation down to the distal lung. Elevated CANO in the presence of eosinophilia is clinically relevant because distal inflammation has been linked to poor asthma control, frequent exacerbations, and air trapping, even when FEV_1_ is preserved [6,43]. Thus, the positive association between Eos and CANO in our cohort suggests that patients with high systemic eosinophil counts may harbor clinically meaningful peripheral lung inflammation that warrants targeted assessment using partitioned NO measurement.

Our results suggest that, in addition to their relationship with exhaled NO parameters, elevated Eos levels were associated with lower FEF_25–75_, FEF_75_, and FEV_1_, as well as higher RV and RV/TLC. This pattern is indicative of reduced airflow throughout the airways and air trapping, hallmark features of obstructive inflammatory airway diseases. These observations are in line with previous reports [44,45]. Taken together with the associations between systemic Eos and exhaled NO parameters discussed above, these findings suggest that exhaled NO markers may also be closely related to small airway ventilatory indices characteristic of SAD, as well as to FEV_1_, a conventional spirometric index of overall airflow limitation in asthma.

When examining this possibility, we observed an inverse association of CANO with FEF_25–75_ and FEF_75_, but not with FEV_1_, whereas the proximal inflammatory markers (FeNO and J’awNO) showed no significant relationship with these spirometric indices. These findings support the notion of compartmentalized airway inflammation in asthma and suggest that CANO can reveal peripheral lung impairment not captured by traditional proximal indicators. Prior studies using models that account for axial diffusion have reported significant correlations between distal NO measures and mid-to-late expiratory flows (for example, FEF_25–75_) but not with FEV_1_/FVC or sputum eosinophils [37,46]. Other reports have shown strong negative correlations between CANO and FEF_75_ or FEF_50_ in patients with asthma or asthma-like symptoms despite normal conventional lung function indices, suggesting that increasing distal inflammation is associated with declining small airway patency [11,46]. Beyond spirometry, the association between CANO and SAD has been supported by other PFTs, including body plethysmography, IOS, and multiple breath nitrogen washout [38,47]. This was indeed shown in our study, in which CANO was positively associated with RV and RV/TLC, two plethysmographic indices reflecting SAD [9].

The physiological link between CANO and FEF_25–75_ or FEF_75_ is consistent with pathological observations in severe asthma: histological studies show that small airways are major sites of inflammation and remodeling, with dense inflammatory cell infiltration extending from the bronchioles toward alveolar tissue [4,48,49]. If distal inflammation is not adequately controlled, it may progress to persistent SAD [3,50]. Because the distal airways have a large total cross-sectional area, substantial obstruction can develop in this “quiet zone” with minimal impact on FEV_1_ or total airway resistance, which may explain why CANO tracks with FEF_25–75_ and FEF_75_ (but not with FEV_1_), while proximal NO markers do not [4,7,51]. Whereas FEF_25–75_ and FEF_75_ reflect SAD by detecting distal airflow limitation, RV and RV/TLC represent some of the earliest manifestations of SAD, arising from premature airway closure, where small airways collapse at higher lung volumes before expiration is complete, leading to air trapping or lung hyperinflation [17,18].

Clinically, the associations between CANO and spirometric and plethysmographic indices help explain discordant patterns in which CANO is elevated while FeNO remains normal. ICS effectively suppress proximal airway inflammation by reducing iNOS expression (lowering FeNO and J’awNO) but may incompletely treat the distal lung because peripheral deposition is limited by aerosol particle size and airway geometry [52,53,54]. Prior studies have reported isolated CANO elevation in symptomatic patients despite standard assessments and higher CANO in more severe or refractory asthma even with high-dose ICS, suggesting that the distal compartment can remain a reservoir of ongoing inflammation associated with reduced distal expiratory flows such as FEF_25–75_ and FEF_75_ [25,47]. Mechanistically, higher CANO in the setting of peripheral obstruction may reflect both increased NO production from inflamed small airway or alveolar regions and altered NO transport dynamics (including axial diffusion), which can amplify distal NO signals when small airway narrowing is present [11,26,37,55]. Together, these data suggest that CANO can complement FeNO, spirometry, and plethysmography as a biomarker for identifying a small airway phenotype that may require targeted therapeutic strategies.

Our study has several limitations. First, CANO was calculated using the standard two-compartment model, which does not account for axial diffusion from the conducting airways to the alveolar region and may therefore overestimate CANO in some circumstances [26,37,56]. Second, SAD was assessed using spirometric indices, which are effort-dependent and more variable than other small-airway-specific techniques such as IOS or multiple breath nitrogen washout; these methods were not available in our study [9,51]. Third, we used Eos as a systemic marker of T2 inflammation; although correlations were significant, we lacked invasive measures (BAL or transbronchial biopsies) to directly confirm eosinophilic infiltration in distal lung tissue, and the 3-month window between blood sampling and functional testing may have introduced temporal variability in Eos values [25,48]. Fourth, although all patients were receiving ICS therapy, and the adjusted models included antihistamine and anti-leukotriene use, the absence of reliable data on ICS dose, oral corticosteroid exposure, and biologic therapy may have resulted in residual confounding related to treatment intensity. Finally, the cross-sectional design precludes causal inference regarding the role of ICS treatment in the observed associations and whether distal inflammation drives progression of small airway obstruction over time.

## 5. Conclusions

In this cohort, blood eosinophil levels were independently and consistently associated with exhaled NO indices across airway compartments. Only the distal NO component (CANO) showed significant associations with small airway and air trapping indices, supporting a link between distal airway inflammation and SAD in asthma. These findings suggest that partitioned multi-flow exhaled NO measurements may complement standard FeNO and spirometry by capturing distal inflammatory activity relevant to small airway involvement. Future prospective studies should assess whether partitioned exhaled NO parameters, combined with small airway functional tests, can improve identification and monitoring of SAD in both high- and low-T2 asthma phenotypes.

## Figures and Tables

**Table 1:** **Characteristics of the study population.**

Parameters	Unit	Distribution Characteristics
Age	years	54.23 (15.99)
Height	cm	167.10 (9.28)
BMI	kg/m^2^	24.6 [22.10; 28.00]
Eosinophil count		260 [110; 540]
**Exhaled nitric oxide**		
FeNO (50 mL/s)	ppb	23.61 [15.36; 52.97]
FeNO (100 mL/s)	ppb	14.69 [9.30; 33.30]
FeNO (150 mL/s)	ppb	11.42 [7.20; 23.64]
J’awNO	nL/min	60.20 [38.40; 141.20]
CANO	ppb	3.80 [2.50; 6.62]
**Forced spirometry**		
FEV_1_ z-score		−1.49 (1.46)
FVC z-score		−0.92 (1.31)
FEV_1_/FVC z-score		−1.16 (1.27)
FEF_25–75_ z-score		−1.27 (1.29)
FEF_75_ z-score		−0.62 (0.96)
**Plethysmography**		
TLC z-score		0.29 (1.22)
FRC z-score		0.76 (0.96)
RV z-score		1.26 (0.94)
RV/TLC z-score		1.60 (1.10)
VC z-score		−0.95 (1.36)

***Note:*** *Data are presented as mean (SD) or median [IQR] as appropriate, according to variable distribution. BMI, body mass index; FeNO, fractional exhaled nitric oxide; J’awNO, maximal bronchial nitric oxide flux; CANO, alveolar nitric oxide concentration; FEV_1_, forced expiratory volume in 1 s; FVC, forced vital capacity; FEF_25–75_, forced expiratory flow between 25% and 75% of FVC; FEF_75_, forced expiratory flow at 75% of FVC; TLC, total lung capacity; FRC, functional residual capacity; RV, residual volume; VC, vital capacity. Z-scores are expressed for PFT variables relative to GLI reference equations, with lower values indicating lower-than-predicted function and higher values indicating higher-than-predicted values relative to the reference population.*

**Table 2:** **Associations among eosinophil count (log scale, per unit), exhaled NO levels, and lung function indices.**

Response Variables	Unadjusted Marginal Slope			Adjusted Marginal Slope		
	Estimate	95% CI	*p* Value #	Estimate	95% CI	*p* Value #
**FeNO 50 mL/s (ppb)**	15.724	12.670–18.778	<0.0001	12.107	9.449–14.764	<0.0001
**FeNO 100 mL/s (ppb)**	7.889	6.178–9.601	<0.0001	6.326	4.823–7.829	<0.0001
**FeNO 150 mL/s (ppb)**	6.278	5.102–7.454	<0.0001	4.860	3.842–5.878	<0.0001
**J’awNO (nL/min)**	43.298	34.249–52.348	<0.0001	33.556	25.698–41.413	<0.0001
**CANO (ppb)**	1.675	1.332–2.018	<0.0001	1.136	0.842–1.430	<0.0001
**RV z-score**	0.135	0.075–0.196	<0.0001	0.136	0.075–0.196	<0.0001
**RV/TLC z-score**	0.107	0.035–0.179	0.0035	0.128	0.056–0.201	0.0005
**FEF_25–75_ z-score**	−0.168	−0.251; −0.086	<0.0001	−0.169	−0.253; −0.085	<0.0001
**FEF_75_ z-score**	−0.171	−0.233; −0.110	<0.0001	−0.169	−0.230; −0.109	<0.0001
**FEV_1_ z-score**	−0.193	−0.286; −0.100	<0.0001	−0.201	−0.295; −0.106	<0.0001

***Note:** The estimand was the raw or adjusted average marginal slope, defined as the average partial derivative of the model-based expected outcome with respect to log eosinophil count across the study population. Thus, each estimate represents the absolute change in the response variable associated with a one-unit increase in log(eosinophil count). # Adjusted marginal slopes were estimated from regression models including sex, age, and body mass index as covariates. For exhaled NO outcomes, active smoking status, anti-leukotriene therapy, and antihistamine use were additionally included as covariates. Exhaled NO outcomes were modeled using a Gamma distribution; lung function outcomes expressed as z-scores were modeled using a Gaussian distribution. Log-transformed eosinophil count was modeled using a quadratic polynomial term to allow for potential non-linearity. CI, confidence interval; FeNO, fractional exhaled nitric oxide; J’awNO, maximal bronchial nitric oxide flux; CANO, alveolar nitric oxide concentration; FEF_25–75_, forced expiratory flow between 25% and 75% of FVC; FEF_75_, forced expiratory flow at 75% of FVC; FEV_1_, forced expiratory volume in 1 s; RV, residual volume; TLC, total lung capacity. # p values are two-sided and test the null hypothesis that the adjusted average marginal slope equals 0. Results were considered statistically significant at p < 0.05.*

**Table 3:** **Association between alveolar nitric oxide level and lung function indices.**

Response Variables	Unadjusted Marginal Slope			Adjusted Marginal Slope #		
	Estimate	95% CI	*p* Value	Estimate	95% CI	*p* Value
**RV z-score**	0.113	0.073; 0.152	<0.001	0.106	0.065; 0.146	<0.001
**RV/TLC z-score**	0.059	0.012; 0.106	0.013	0.065	0.016; 0.115	0.009
**FEF_25–75_ z-score**	−0.058	−0.112; −0.004	0.036	−0.078	−0.135; −0.020	0.008
**FEF_75_ z-score**	−0.109	−0.171; −0.046	<0.001	−0.156	−0.229; −0.082	<0.001
**FEV_1_ z-score**	−0.030	−0.091; 0.031	0.340 (NS)	−0.042	−0.107; 0.022	0.200 (NS)

***Note:** The estimand was the raw or adjusted average marginal slope, defined as the average partial derivative of the model-based expected outcome with respect to CANO across the study population. # Adjusted marginal slopes were estimated from Gaussian regression models for z-score outcomes, with adjustment for sex, age, and body mass index. CANO was modeled using a quadratic polynomial term to allow for potential non-linearity. Accordingly, each estimate represents the adjusted absolute change in the lung function z-score associated with a 1 ppb increase in CANO. CI, confidence interval; CANO, alveolar nitric oxide concentration; FEF_25–75_, forced expiratory flow between 25% and 75% of FVC; FEF_75_, forced expiratory flow at 75% of FVC; FEV_1_, forced expiratory volume in 1 s; RV, residual volume; TLC, total lung capacity. p values are two-sided and test the null hypothesis that the adjusted average marginal slope equals 0, at the significance threshold of p < 0.05.*

## Data Availability

The data presented in this study are available on request from the corresponding author. The data are not publicly available due to privacy and ethical restrictions related to patient confidentiality.

## Supplementary Materials

The following supporting information can be downloaded at: https://www.mdpi.com/article/10.3390/medsci14020292/s1, Supplementary methodology; Table S1: Distribution of categorized PFT outcomes; Table S2: Association between log-transformed eosinophil count and exhaled NO levels or small airway ventilatory function indices, conditioned by eosinophil level; Table S3: Association between J’awNO level and small airway ventilatory function indices; Table S4: Association between FeNO level and small airway ventilatory function indices; Figure S1: Distribution of exhaled nitric oxide parameters.
