# Supplementary material for: Distal Airway Inflammation Is Linked to Small Airway Dysfunction in Asthma
Source: Med Sci (Basel). 2026 Jun 5;14(2):292. doi: 10.3390/medsci14020292 (PMC13302983; doi:10.3390/medsci14020292)
Supplement: Supplementary file 1 [file medsci-14-00292-s001.zip › medsci-4333604-supplementary/Supplementary files/Supplementary methodology.pdf]

Supplementary methodology

Sample size justification

A simulation procedure was conducted on retrospective data to justify the sample size is sufficient to detect a statistically significant association between eosinophil count level and alveolar NO (CANO), as well as between FEF25-75 z-score and CANO, using the same modeling framework as in the primary analysis. The effect of interest was defined as the average marginal slope (dy/dx) of log(Eos) or CANO.

For each simulated dataset, a regression model was fitted, for example:

$$\text{CANO} \sim \text{poly}(\text{Log\_Eos}, 2) + \text{AGE} + \text{BMI} + \text{SEX}$$

using a Gamma family for CANO, and Normal family for FEF25-75 z-score. After model fitting, the average marginal slope (dy/dx) for Log_Eos or CANO was estimated using the average_slopes() function from the *marginal effects* package.

A simulation-based resampling procedure was used to assess empirical power across a range of candidate sample sizes. Specifically, random samples of size n were repeatedly drawn from mod_df without replacement. The sample sizes evaluated were:

$$n = 10,20,30,\ldots,200$$

For each sample size, the following steps were performed:

1. A random subsample of size n was drawn from original dataset;
2. The predefined GAMLSS model was fitted to the subsample.
3. The average marginal slope (dy/dx) for Log_Eos or CANO was estimated.
4. The slope was considered statistically significant if the associated p-value was below 0.05.
5. Steps 1–4 were repeated 100 times for the same sample size.

The empirical power at each sample size was defined as the proportion of the 100 simulation replicates in which the slope was statistically significant. Therefore, the power estimate for each n can be written as:

$$\text{Power}(n) = \frac{\text{Number of replicates with } p < 0.05}{100}$$

The results of simulation procedure are represented as power curves:

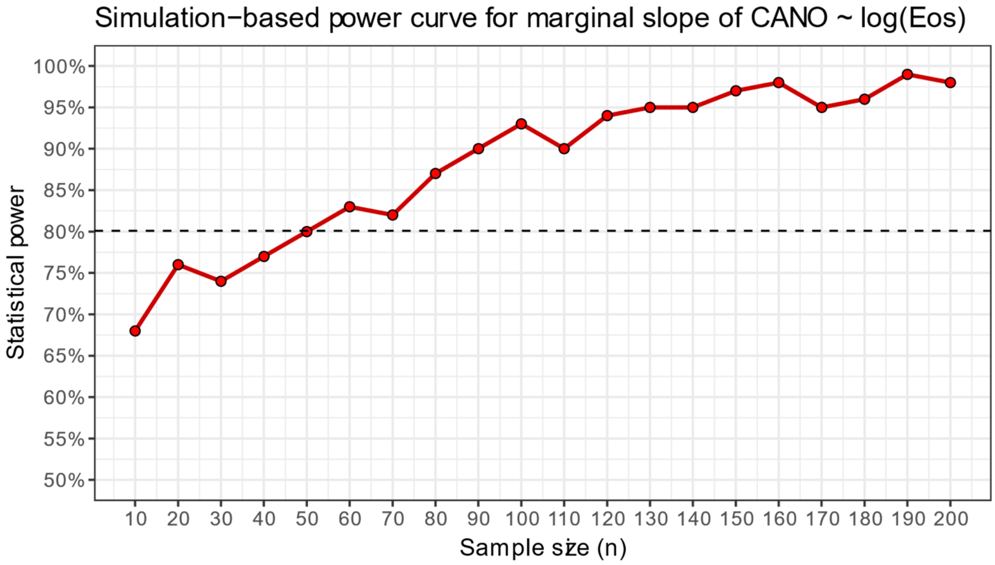

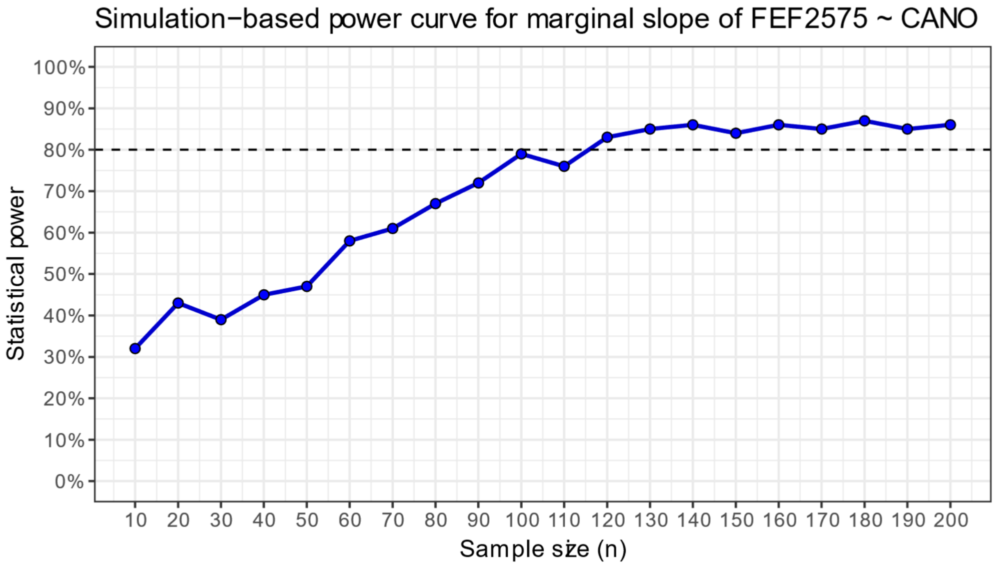

These power curve provide a visual summary of how the probability of detecting a statistically significant average slope changes as the sample size increases.

According to these results, a sample size of at least 100 patients is sufficient for achieving a significative marginal slope for CANO and Eosinophil count with statistical power ≥ 80%.

Exhaled nitric oxide measurement

Exhaled nitric oxide was measured before spirometry using the HypAir FeNO device (Medisoft/MGC Diagnostics, Sorinnes, Belgium). After inhalation of NO-free air to total lung capacity, participants performed controlled exhalations against a positive mouth pressure to limit nasal NO contamination and to maintain stable target expiratory flows. Measurements were obtained at 50, 100, and 150 mL/s. At each flow rate, at least two technically acceptable measurements were required, with a coefficient of variation below 10%; when this criterion was not met, an additional maneuver was performed. The mean plateau NO concentration at each flow was used for subsequent calculation.

CANO and J'awNO were derived using the standard two-compartment model of pulmonary NO exchange. This model conceptualizes the lung as two functional NO-producing compartments: a proximal conducting airway compartment, represented by the maximal bronchial NO flux, J'awNO, and a distal/alveolar compartment, represented by the alveolar NO concentration, CANO. For each expiratory flow, NO output was calculated as: V'NO = FeNO × V'E, where V'NO is the exhaled NO output, FeNO is the measured exhaled NO concentration at the corresponding flow, and V'E is the expiratory flow. After conversion of expiratory flow to L/min, NO output is expressed in nL/min. The standard linear model is then defined as: V'NO = J'awNO + CANO × V'E.

In this framework, the y-intercept of the linear relationship between NO output and expiratory flow estimates J'awNO, expressed in nL/min, whereas the slope estimates CANO, expressed in ppb. These calculations were performed automatically by the manufacturer's software; no external post-processing algorithm was used.

Axial diffusion correction was not applied. The standard two-compartment model does not explicitly account for axial back-diffusion of NO from the conducting airways toward the alveolar region, which may lead to overestimation of absolute CANO values in some circumstances. However, axial diffusion correction requires a more complex model and multiple measurements at higher expiratory flows, typically above 50 mL/s and extending over approximately 100–350 mL/s. Such measurements are not always feasible in routine clinical patients and were not available through the automated software workflow used in this study. We therefore used the standard two-compartment model based on three clinically feasible sampling flows, 50, 100, and 150 mL/s. This choice was made to ensure feasibility, reproducibility, and consistency with routine clinical practice.

Statistical analysis procedure

Statistical objectives, clinical context, and hypotheses

The main analysis aims to evaluate the associations between markers of small-airway inflammation and two clinically relevant domains. The first domain consists of the association between blood eosinophil count on the logarithmic scale, and exhaled nitric oxide (NO) markers, (FeNO50, FeNO100, FeNO150, J'awNO, and CANO). The second domain consists of the association between these inflammatory biomarkers, including log-transformed eosinophil count and exhaled nitric oxide indices, and lung function testing-derived markers of small-airway obstruction, namely FEF25–75, FEF75, RV, and RV/TLC, each analyzed on the z-score scale.

The estimand is the adjusted average marginal slope of the expected outcome with respect to the predictor of interest, after adjustment for relevant covariates including sex, age, and body mass index. Because the associations are nonlinear, especially for log eosinophil count and exhaled NO, the analysis is based on nonlinear regression models with second-degree polynomial terms.

For the first objective, let $Y_i^{(B)}$denote a biomarker outcome for participant i, where $Y_i^{(B)} \in \{\text{FeNO50,FeNO100,FeNO150,J'awNO,CANO}\}$, and let $X_i = \log(\text{Eosinophil}_i)$. The scientific question is whether, on average, the expected biomarker level changes as eosinophil burden increases. The null and alternative hypotheses are therefore expressed in terms of the marginal slope: $H_0: \theta = 0$ versus $H_1: \theta \neq 0$; where θ is the adjusted average marginal slope of the expected biomarker level with respect to log eosinophil count.

For the second objective, let $Y_i^{(Z)}$denote a distal airway functional parameter on the z-score scale, where $Y_i^{(Z)} \in \{\text{FEF25-75,FEF75,RV,RV/TLC}\}$, and let X_i denote the inflammatory predictor under study, such as log eosinophil count, CANO, or another exhaled NO biomarker. The corresponding hypothesis is $H_0: \theta = 0$ versus $H_1: \theta \neq 0$; where θ is now the adjusted average marginal slope of the expected z-score outcome with respect to the selected inflammatory predictor.

Distributional assumptions and regression model structure

The analysis is implemented through generalized linear models (GLM). The general model structure may be written as

$$g\{\mu_i\} = \eta_i = \beta_0 + f(X_i) + \gamma_1 \text{Age}_i + \gamma_2 \text{BMI}_i + \gamma_3 \text{Sex}_i,$$

where $\mu_i = E(Y_i \mid X_i, \mathbf{C}_i)$ is the conditional mean of the outcome for participant i, $g(\cdot)$ is the link function associated with the chosen outcome distribution, X_i is the predictor of interest, $f(X_i)$ is the prespecified functional form for that predictor, and $\mathbf{C}_i$ denotes the covariate vector. The term β_0 is the intercept, and $\gamma_1, \gamma_2, \gamma_3$ are regression parameters quantifying covariate adjustment.

When the predictor of interest is log eosinophil count or CANO, the association is modeled nonlinearly using a quadratic polynomial. Conceptually, this can be written as

$$f(X_i) = \beta_1 X_i + \beta_2 X_i^2.$$

For biomarker outcomes such as FeNO50, FeNO100, FeNO150, J'awNO, and CANO, a Gamma distribution is appropriate because these outcomes are strictly positive and often right-skewed. A standard way to describe this model is

$$Y_i^{(B)} \mid X_i, \mathbf{C}_i \sim \text{Gamma}(\mu_i, \phi),$$

where $\mu_i > 0$ is the conditional mean and $\phi$is a dispersion parameter.

For the distal airway parameters analyzed on the z-score scale, a Gaussian model is used:

$$Y_i^{(Z)} \mid X_i, \mathbf{C}_i \sim \mathcal{N}(\mu_i, \sigma^2),$$

where μ_i is the conditional mean z-score and σ^2 is the residual variance.

The adjusted average marginal slope is defined from the conditional mean function. For an individual participant i, the marginal slope with respect to predictor $X_i$is

$$s_i = \frac{\partial \mu_i}{\partial X_i}.$$

The target estimand is the sample-average marginal slope:

$$\theta = \frac{1}{n} \sum_{i=1}^{n} \frac{\partial \mu_i}{\partial X_i}.$$

The derivative can also be expressed more generally through the chain rule:

$$\frac{\partial \mu_i}{\partial X_i} = \frac{d\, g^{-1}(\eta_i)}{d\eta_i} \cdot \frac{d\, f(X_i)}{dX_i}.$$

Statistical inference framework and clinical meaning

All hypothesis tests will be two-sided, and statistical significance will be defined using a prespecified threshold of $p<0.05$. A result will be regarded as statistically significant when the two-sided p-value < 0.005, or equivalently when the corresponding 99.5% confidence interval excludes 0.

The point estimate of the average marginal slope should be interpreted on the natural scale of the outcome. For biomarker outcomes modeled with the Gamma family, the estimand represents the adjusted average change in the expected biomarker level associated with a one-unit increase in the predictor. When the predictor is log eosinophil count, a one-unit increase corresponds to an e-fold increase in eosinophil count. Therefore, a positive slope indicates that higher eosinophilic burden is associated with higher expected exhaled NO biomarker levels, whereas a negative slope indicates the opposite. Because the

association is nonlinear, the average marginal slope should be understood as an average local rate of change across the observed participant distribution rather than as a globally constant increment.

For clinical interpretation, a positive adjusted average marginal slope linking log eosinophil count to FeNO50, FeNO100, FeNO150, J'awNO, or CANO would support the hypothesis that greater systemic eosinophilic inflammation is associated with greater airway inflammatory activity as reflected by exhaled nitric oxide. In particular, positive associations involving J'awNO or CANO may be clinically relevant because these indices are often interpreted as markers more closely related to peripheral or distal airway inflammatory processes. Conversely, an estimate close to zero with narrow confidence limits would suggest little evidence of a clinically meaningful monotonic association in the observed population.

For z-score outcomes, the average marginal slope quantifies the adjusted mean change in the z-score of a physiological parameter per unit increase in the inflammatory predictor. The clinical direction of interpretation depends on the outcome. For FEF25–75 and FEF75, lower z-scores indicate poorer distal expiratory flow; therefore, a negative marginal slope implies that higher inflammatory burden is associated with worse small-airway flow limitation. For RV and RV/TLC, higher z-scores indicate greater gas trapping or hyperinflation; therefore, a positive marginal slope implies that higher inflammatory burden is associated with more severe distal airway dysfunction. These directional interpretations are central for translating statistical results into clinically meaningful conclusions.

Clinical significance concerns whether the magnitude of the estimated association is large enough to matter in practice, whether it is biologically plausible, and whether it is consistent with current pathophysiological understanding. For example, a very small slope may reach statistical significance in a large sample yet be of limited clinical relevance. Conversely, a clinically meaningful slope with imprecise estimation may fail to cross the $p < 0.05$ threshold, in which case the result should be described as suggestive but inconclusive rather than negative in an absolute sense.

Interpretation of each model should integrate four elements simultaneously: the sign of the estimate, the magnitude of the average marginal slope, the precision of the estimate as reflected by its confidence interval, and the clinical directionality of the underlying outcome. Inflammatory predictors associated with lower FEF25–75 or FEF75 and higher RV or RV/TLC would collectively support the biological interpretation that eosinophilic or nitric oxide–defined inflammation tracks with distal airway dysfunction. However, because the study is cross-sectional, such findings should be described as consistent with, rather than proving, a mechanistic relationship.

Finally, all reported results should be phrased in terms of adjusted expected mean differences on the outcome scale induced by incremental changes in the predictor, averaged over the study population.
