# Supplementary material for: Distal Airway Inflammation Is Linked to Small Airway Dysfunction in Asthma
Source: Med Sci (Basel). 2026 Jun 5;14(2):292. doi: 10.3390/medsci14020292 (PMC13302983; doi:10.3390/medsci14020292)
Supplement: Supplementary file 1 [file medsci-14-00292-s001.zip › medsci-4333604-supplementary/Supplementary files/Supplementary tables.pdf]

Table S1: Distribution of categorized PFT outcomes

Parameters	Rule	Frequency (%)
**Spirometry patterns**		
Normal	ATS-ERS	61 (50.4 %)
Obstruction	ATS-ERS	24 (19.8 %)
Possible mixed disorder	ATS-ERS	17 (14.1 %)
Possible restriction or non-specific	ATS-ERS	19 (15.7 %)
**Forced spirometry indices**		
Low FEV_1	FEV1 z-score < -1.645	58 (47.9 %)
FEV_1/FVC < 70%	FEV1/FVC ratio < 0.7	55 (45.5 %)
Low FEF_{25-75}	FEF_{25-75} z-score < -1.645	52 (43.0 %)
Low FEF_{75}	FEF_{75} z-score < -1.645	21 (17.4 %)
**Residual volume**		
High RV	RV z-score > 1.645	33 (27.3 %)
High RV/TLC	RV/TLC z-score > 1.645	52 (43.0 %)

***Note**: Spirometric patterns were classified according to ATS/ERS interpretive rules. Low spirometric values were defined as z-scores below the lower limit of normal, corresponding to z-score < −1.645. High plethysmographic indices were defined as z-scores above the upper limit of normal, corresponding to z-score > +1.645. FEV_1, forced expiratory volume in 1 second; FVC, forced vital capacity; $FEF_{25–75}$, forced expiratory flow between 25% and 75% of FVC; FEF_{75}, forced expiratory flow at 75% of FVC; RV, residual volume; TLC, total lung capacity.*

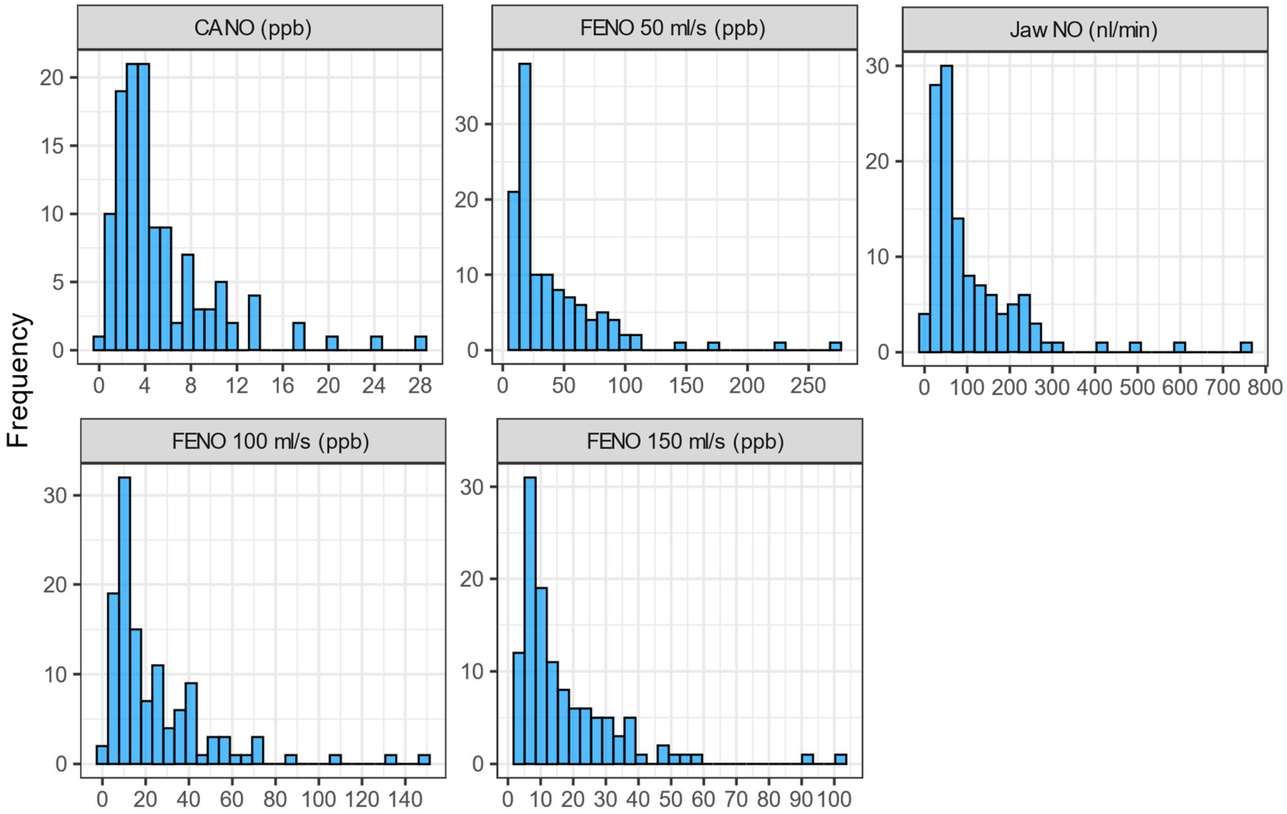

Figure S1: Distribution of exhaled nitric oxide parameters

Table S2: Association between log-transformed eosinophil count and exhaled NO levels or small airway ventilatory function indices, conditioned by eosinophil level

Response variables	Eos range	Unadjusted marginal slope			Adjusted marginal slope*		
		Estimate	95%CI	P value	Estimate	95%CI	P value#
**FeNO 50 ml/s (ppb)**	Low	4.361	2.376 – 6.345	<0.0001	4.566	2.786 – 6.346	<0.0001
	High	28.305	21.965 – 34.646	<0.0001	20.456	14.964 – 25.947	<0.0001
**FeNO 100 ml/s (ppb)**	Low	2.784	1.591 – 3.977	<0.0001	2.763	1.715 – 3.810	<0.0001
	High	13.542	10.087 – 16.996	<0.0001	10.270	7.237 – 13.304	<0.0001
**FeNO 150 ml/s (ppb)**	Low	1.650	0.869 – 2.431	<0.0001	1.640	0.940 – 2.341	<0.0001
	High	11.319	8.933 – 13.705	<0.0001	8.368	6.308 – 10.427	<0.0001
**J'awNO (nl/min)**	Low	12.182	6.423 – 17.942	<0.0001	13.456	8.270 – 18.642	<0.0001
	High	77.749	58.798 – 96.699	<0.0001	55.809	39.439 – 72.179	<0.0001
**CANO (ppb)**	Low	0.319	0.077 – 0.561	0.0097	*0.231*	*0.004 – 0.457*	*0.046 (NS)*
	High	3.177	2.508 – 3.846	<0.0001	2.138	1.567 – 2.709	<0.0001
**RV z-score**	Low	0.010	-0.062 ; 0.082	0.788 (NS)	0.061	-0.010 ; 0.132	0.092 (NS)
	High	0.272	0.162 ; 0.382	<0.0001	0.276	0.166 ; 0.385	<0.0001
**RV/TLC z-score**	Low	0.125	0.040 ; 0.210	0.0041	0.167	0.082 ; 0.252	0.0001
	High	0.087	-0.043 ; 0.218	0.190 (NS)	0.175	0.044 ; 0.305	0.0087
**FEF_{25-75} z-score**	Low	-0.245	-0.344 ; -0.146	<0.0001	-0.289	-0.386 ; -0.192	<0.0001
	High	-0.083	-0.235 ; 0.069	0.283 (NS)	-0.096	-0.246 ; 0.053	0.206 (NS)
**FEF_{75} z-score**	Low	-0.191	-0.264 ; -0.117	<0.0001	-0.189	-0.261 ; -0.118	<0.0001
	High	-0.150	-0.262 ; -0.037	0.0091	-0.200	-0.310 ; -0.090	0.0003
**FEV_1 z-score**	Low	-0.341	-0.452 ; -0.230	<0.0001	-0.357	-0.466 ; -0.248	<0.0001
	High	-0.033	-0.202 ; 0.137	0.705 (NS)	-0.131	-0.298 ; 0.036	0.125 (NS)

Note**:** *The estimand was the raw or adjusted average marginal slope, defined as the average partial derivative of the model based expected outcome with respect to log eosinophil count, estimated separately at two Eosinophile range (high: Eos count > 300). Thus, each estimate represents the absolute change in the response variable associated with a 1-unit increase in log(eosinophil count) within each interval. # Adjusted marginal slopes were estimated from regression models including sex, age, body mas index, anti-inflammarory treatments and active smoking status as covariates.*

Exhaled nitric oxide outcomes were modeled using a Gamma distribution; lung function outcomes expressed as z-scores were modeled using a Gaussian distribution. Log-transformed eosinophil count was modeled using a quadratic polynomial term to allow for potential non-linearity. CI, confidence interval; FeNO, fractional exhaled nitric oxide; J'awNO, maximal bronchial nitric oxide flux; CANO, alveolar nitric oxide concentration; $FEF_{25–75}$, forced expiratory flow between 25% and 75% of FVC; FEF_{75}, forced expiratory flow at 75% of FVC; FEV_1, forced expiratory volume in 1 second; RV, residual volume; TLC, total lung capacity. # p values are two-sided and test the null hypothesis that the adjusted average marginal slope equals 0. Results were considered statistically significant at $p < 0.05$.

Table S3: Association between J'awNO level and small airway ventilatory function indices

Response variables	Unadjusted marginal slope			Adjusted marginal slope #		
	Estimate	95%CI	P value	Estimate	95%CI	P value
**RV**	0.001	-0.000 ; 0.003	0.099	0.001	-0.001 ; 0.002	0.526
**RV/TLC**	<-0.001	-0.002 ; 0.002	0.839	<-0.001	-0.002 ; 0.002	0.969
**FEF_{25-75}**	0.001	-0.001 ; 0.003	0.348	0.001	-0.001 ; 0.004	0.293
**FEF_{75}**	0.001	-0.001 ; 0.002	0.277	<0.001	-0.001 , 0.002	0.605
**FEV_1**	0.002	-0.000 ; 0.004	0.105	0.002	-0.001 ; 0.005	0.254

Note: *CI, confidence interval; J'awNO, maximal bronchial nitric oxide flux; $FEF_{25–75}$, forced expiratory flow between 25% and 75% of FVC; FEF_{75}, forced expiratory flow at 75% of FVC; FEV_1, forced expiratory volume in 1 second; RV, residual volume; TLC, total lung capacity. # p values are two-sided and test the null hypothesis that the adjusted average marginal slope equals 0. Results were considered statistically significant at $p < 0.05$.*

Table S4: Association between FeNO level and small airway ventilatory function indices

Response variables	Unadjusted marginal slope			Adjusted marginal slope #		
	Estimate	95%CI	P value	Estimate	95%CI	P value
**RV**	0.004	-0.000 ; 0.008	0.076	0.003	-0.001 ; 0.008	0.124
**RV/TLC**	<0.001	-0.005 ; 0.004	0.868	<0.001	-0.005 ; 0.005	0.965
**FEF_{25-75}**	0.003	-0.003 ; 0.008	0.361	0.003	-0.003 ; 0.009	0.365
**FEF_{75}**	0.002	-0.002 ; 0.007	0.265	0.002	-0.002 ; 0.006	0.391
**FEV_1**	0.005	-0.001 ; 0.012	0.103	0.005	-0.001 ; 0.012	0.101

Note: *CI, confidence interval; FeNO, fractional exhaled nitric oxide; $FEF_{25–75}$, forced expiratory flow between 25% and 75% of FVC; FEF_{75}, forced expiratory flow at 75% of FVC; FEV_1, forced expiratory volume in 1 second; RV, residual volume; TLC, total lung capacity. # p values are two-sided and test the null hypothesis that the adjusted average marginal slope equals 0. Results were considered statistically significant at $p < 0.05$.*
